# Anti-EGFR monoclonal antibodies and EGFR tyrosine kinase inhibitors as combination therapy for triple-negative breast cancer

**DOI:** 10.18632/oncotarget.12037

**Published:** 2016-09-15

**Authors:** Abderrahim El Guerrab, Mahchid Bamdad, Fabrice Kwiatkowski, Yves-Jean Bignon, Frédérique Penault-Llorca, Corinne Aubel

**Affiliations:** ^1^ Centre Jean Perrin - ERTICa-EA4677, BP392, 63011 Clermont-Ferrand Cedex, France; ^2^ Clermont Université - Université d'Auvergne - ERTICa-EA4677, Faculté de Médecine, BP38, 63001 Clermont-Ferrand Cedex, France; ^3^ Clermont Université - Université d'Auvergne - ERTICa-EA4677, Institut Universitaire de Technologie, Département Génie Biologique, Ensemble Universitaire des Cézeaux, BP86, 63172 Aubière Cedex, France

**Keywords:** triple-negative breast cancer, epidermal growth factor receptor, anti-EGFR targeted therapy, cytotoxicity, cell cycle

## Abstract

Triple-negative breast cancer (TNBC) is characterized by overexpression of epidermal growth factor receptor (EGFR) and activation of its downstream signaling pathways. Dual targeting of EGFR using one monoclonal antibody (mAb; cetuximab or panitumumab) and one tyrosine kinase inhibitor (EGFR-TKI; gefitinib or erlotinib) is a potential therapeutic approach. We investigated the effect of these therapies in EGFR-expressing TNBC cell lines that do or do not harbor the main activating mutations of EGFR pathways. Cell lines were sensitive to EGFR-TKIs, whereas mAbs were active only in MDA-MB-468 (*EGFR* amplification) and SUM-1315 (*KRAS* and *PTEN* wild-type) cells. MDA-MB-231 (*KRAS* mutated) and HCC-1937 (*PTEN* deletion) cells were resistant to mAbs. The combined treatment resulted in a synergistic effect on cell proliferation and superior inhibition of the RAS/MAPK signaling pathway in mAb-sensitive cells. The anti-proliferative effect was associated with G1 cell cycle arrest followed by apoptosis. Sensitivity to therapies was characterized by induction of positive regulators and inactivation of negative regulators of cell cycle. These results suggest that dual EGFR inhibition might result in an enhanced antitumor effect in a subgroup of TNBC. The status of *EGFR*, *KRAS* and *PTEN* could be used as a molecular marker for predicting the response to this therapeutic strategy.

## INTRODUCTION

Triple-negative breast cancer (TNBC) is an aggressive type of cancer that represents approximately 15-20% of invasive breast carcinomas [1, 2]. TNBC is usually identified by reduced expression of estrogen, progesterone and HER2 receptors [3]. This type of tumor is characterized by pejorative clinical outcome with a shorter overall survival than other subtypes of breast cancers. This poor prognosis is due to a lack of therapeutic options. The most effective treatment currently available is based on systemic chemotherapies using anthracycline, taxane and cisplatin [4, 5]. Despite the effectiveness of these therapies, TNBC outcome remains worse with early relapses [6]. In contrast to many other types of cancer, no targeted therapy is currently approved for TNBC.

Approximately 75% of TNBC belong to the basal-like subtype according to molecular classification of Sorlie *et al.* and share a great similarity with infiltrating carcinomas carrying constitutional *BRCA1* mutations [1, 7–10]. These tumors also exhibit chromosomal abnormalities and *p53* mutations [11]. Another feature of TNBC is the overexpression of epidermal growth factor receptor (EGFR) in the majority of cases [3]. EGFR is a transmembrane tyrosine kinase receptor member of the HER family. Autophosphorylation of the intracellular domain of this receptor activates downstream RAS/MAPK and PI3K/AKT pathways that lead to transcriptional regulation of genes involved in cell proliferation, survival and drug resistance [12]. Positive expression of EGFR is associated with poor clinical outcome in several tumor types, including TNBC [13, 14]. Consequently, EGFR is an emerging therapeutic target for the treatment of TNBC.

The two main therapeutic approaches for targeting EGFR rely on the use of monoclonal antibodies (mAbs) and small molecule EGFR tyrosine kinase inhibitors (EGFR-TKIs). Anti-EGFR mAbs target the extracellular domain and EGFR-TKIs competitively block the binding of adenosine 5′ triphosphate to the intracellular catalytic domain of EGFR. In both cases, mAbs and EGFR-TKIs are able to inhibit EGFR activation and thus suppress its downstream signal transduction [15]. Cetuximab and panitumumab are two mAbs that are approved for the treatment of EGFR-expressing metastatic colorectal cancer with *KRAS* wild-type. Gefitinib and erlotinib are two selective EGFR-TKIs used as therapy for patients with advanced or metastatic non-small-cell lung cancer who carry activating *EGFR* mutations [16–18]. Various preclinical and clinical studies have already evaluated the effect of these EGFR inhibitors in combination with conventional cytotoxic chemotherapies in TNBC [19, 20]. Corkery *et al.* have reported an anti-proliferative effect of erlotinib and gefitinib combined with docetaxel or carboplatin in TNBC cell lines [21]. In a randomized phase II study, Baselga *et al.* demonstrated that cisplatin plus cetuximab significantly increased the overall response rate achieved with cisplatin alone in patients with TNBC [22]. Carboplatin has also been reported to be effective in combination with cetuximab [20]. Recently, our group showed the efficacy of cetuximab and panitumumab combined with an anthracycline/taxane-based chemotherapy through multicentric neoadjuvant pilot studies in operable TNBC [23, 24].

As mAbs and EGFR-TKIs target distinct molecular domains of the EGFR, we hypothesized that the combination of these two classes of EGFR inhibitors could be a potential therapeutic strategy for the treatment of EGFR-expressing cancers. However, few studies have investigated the effect of dual targeting of EGFR in TNBC. Huang *et al.* demonstrated that a combination of cetuximab plus gefitinib or erlotinib enhanced growth inhibition and apoptosis of head and neck cancer cell lines over that observed with either agent alone [25]. They also showed that combined treatment significantly inhibited the growth of tumor xenografts from NSCLC cell lines [25]. Other authors have demonstrated in various human cancer cells, including TNBC cell lines, that combination of cetuximab with gefitinib has a synergistic effect on cell proliferation and EGFR downstream signaling pathways [26]. Ferraro *et al.* demonstrated that a cooperative anti-EGFR mAb mixture results in growth inhibition of TNBC cell lines both *in vitro* and *in vivo* [27].

According to the evidence provided by these studies, we investigated the impact of the four main anti-EGFR-targeted therapies on different TNBC cell lines. Based on the hypothesis that the two anti-EGFR strategies (mAbs and EGFR-TKIs) could have complementary mechanisms of action, we studied the effect of two mAbs, cetuximab and panitumumab, and two EGFR-TKIs, erlotinib and gefitinib as single agents and in combination on TNBC cell lines. We analyzed the effects of these therapies on cell viability, EGFR signaling pathways, cell cycle and apoptosis. We also examined the molecular basis for sensitivity and/or resistance to EGFR inhibitors by quantifying the expression of genes involved in RAS/MAPK and PI3K/AKT pathways, cell cycle control, apoptosis, angiogenesis, DNA repair and drug resistance.

## RESULTS

### EGFR signaling pathways are activated in TNBC cell lines

**Figure 1:**
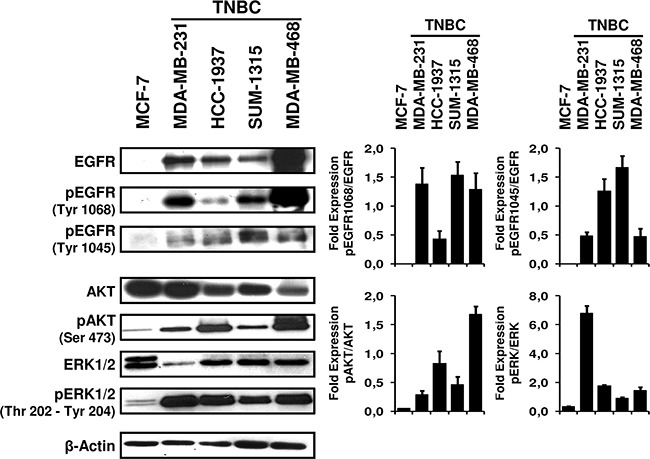
Overexpression of phosphorylated EGFR, AKT and ERK1/2 in TNBC cell lines Basal levels of EGFR, AKT, ERK1/2 and their phosphorylated forms were evaluated by Western blot analysis in TNBC cell lines and the non-TNBC MCF-7 cell line after 24 h of culture. Cells were lysed, and 15 μg of whole cell protein extract was separated by 10% SDS-PAGE and immunoblotted using the indicated antibodies. Beta-actin antibody was used as a loading control. The data shown are representative of two independent experiments. Bar charts depict densitometric quantification of Western blot signals as described in the Materials and Methods.

We evaluated the expression level of total and activated (phosphorylated) forms of EGFR by Western blot (Figure 1). Higher levels of EGFR were detected in TNBC cells compared to the non-TNBC cell line MCF-7, which does not express EGFR. Levels of phosphorylated EGFR were also increased only in TNBC cell lines. The highest and lowest levels of total EGFR expression were observed in the MDA-MB-468 and SUM-1315 cell lines, respectively. The purpose of EGFR autophosphorylation is to activate signaling pathways, such as PI3K/AKT and RAS/MAPK pathways [28]. We next investigated the activation of these pathways by quantifying total and activated (phosphorylated) forms of AKT and ERK1/2 proteins. Increased amounts of phospho-AKT and phospho-ERK1/2 were clearly detected in TNBC cell lines compared to MCF-7 cells. By contrast, expression of total AKT and ERK1/2 were lower in TNBC cell lines than in MCF-7 cells. Based on the level of AKT and ERK phosphorylation, PI3K/AKT and RAS/MAPK signaling pathways were more activated, respectively, in MDA-MB-468 and MDA-MB-231 cell lines, than in others. In the HCC-1937 cell line, increased phosphorylation of both AKT and ERK1/2 was also observed. On the contrary, these pathways were the least activated in the SUM-1315 cell line.

### Differential growth inhibitory effect on TNBC cell lines treated with anti-EGFR monoclonal antibodies or EGFR tyrosine kinase inhibitors as single agents or in combination

**Figure 2:**
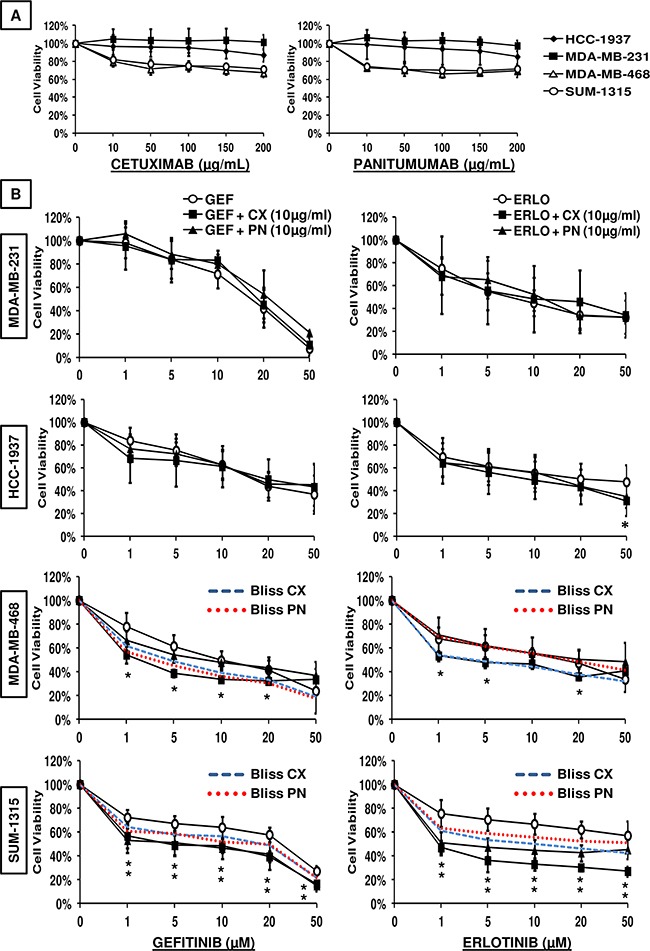
Viability assay of TNBC cell lines treated with anti-EGFR-targeted therapies **A.** Cells were treated for 24 h with increasing concentrations of cetuximab or panitumumab and **B.** with the indicated concentrations of EGFR-TKIs (erlotinib (ERLO) or gefitinib (GEF)) combined with 10 μg/ml anti-EGFR mAbs (cetuximab (CX) or panitumumab (PN)). Cell viability was assessed using the SRB assay as described in the Materials and Methods. The results are expressed as percent of viability of untreated cells and are the mean values ± SEM of three independent experiments. Dashed curves represent the expected Bliss values if the combined effects were additive. The theoretical Bliss curves are shown for mAb-sensitive cell lines (MDA-MB-468 and SUM-1315). *p<0.05 for comparison using two-way analysis of variance (ANOVA) followed by an unpaired Student's t-test between cells treated with EGFR-TKIs and cells treated with combination therapy.

We evaluated the anti-proliferative effect of cetuximab, panitumumab, gefitinib and erlotinib, given alone or in combination, on TNBC cell lines. We first studied the cytotoxic effect of mAbs as single agents (Figure 2A). Cells were treated with increasing concentrations of mAbs for 24 h. Both antibodies inhibited proliferation of MDA-MB-468 and SUM-1315 cell lines from 20 to 30% compared with untreated cells whereas no effect was observed in MDA-MB-231 and HCC-1937 cell lines. The growth inhibitory effect observed in MDA-MB-468 and SUM-1315 was achieved with an antibody concentration of 10 μg/mL and remained stable for higher concentrations. Both MDA-MB-231 and HCC-1937 cell lines appeared to have mechanisms of resistance to anti-EGFR mAbs.

We then tested the associations of mAbs with EGFR-TKIs. Cells were exposed to increasing concentrations of gefitinib or erlotinib as single agents and in combination with a fixed dose of antibodies for 24 h. In light of previous results, the antibody concentration was adjusted to 10 μg/mL. As shown in Figure 2B, single EGFR-TKIs inhibited proliferation of all cell lines in a dose-dependent manner. The half inhibitory concentration values (IC50) of gefitinib and erlotinib as single agents and in combination with cetuximab and panitumumab are summarized in Table 1. The results showed differential effects of gefitinib and erlotinib, which were most active on MDA-MB-468 (IC50 = 10.0 μM) and MDA-MB-231 cells (IC50 = 7.0 μM), respectively. SUM-1315 cells were relatively sensitive to erlotinib alone (although the IC50 was not reached), while gefitinib reduced cell viability from approximately 75% with a concentration of 50 μM.

**Table 1:** IC50 values (μM) of gefitinib and erlotinib as single agents and in combination with anti-EGFR monoclonal antibodies

Cell lines	MDA-MB-231	HCC-1937	MDA-MB-468	SUM-1315
**Gefitinib**	16.5	16.1	10.0	25.3
**Gefitinib + Cetuximab**	18.3	20.0	1.5	4.1
**Gefitinib + Panitumumab**	22.3	17.0	7.9	5.9
**Erlotinib**	7.0	20.0	15.9	-
**Erlotinib + Cetuximab**	8.4	20.0	2.9	1.1
**Erlotinib + Panitumumab**	11.2	14.1	10.0	1.7

In an attempt to evaluate the combined efficacy, the Bliss independence model was used to determine whether the effect of combination therapies was additive, synergistic or antagonistic. When we exposed cells to a combination of EGFR-TKIs with 10 μg/mL of mAbs, no significant decrease of cell viability was observed in MDA-MB-231 and HCC-1937 cells compared with EGFR-TKIs treatment alone. When we compared the experimental with the Bliss theoretical curves, the effect of the combination treatments showed pure additivity for these two cell lines (data not shown). In MDA-MB-468 cells, the addition of cetuximab significantly increased the cytotoxicity effect of both gefitinib (IC50 = 1.5 μM) and erlotinib (IC50 = 2.9 μM) at concentrations ranging from 1 to 20 μM. Cetuximab combined with 5 μM of gefitinib reduced the viability of MDA-MB-468 cells from 60%, while gefitinib alone reduced it from 38%. In the SUM-1315 cell line, both mAbs significantly enhanced the growth inhibitory effect of EGFR-TKIs at all concentrations. Panitumumab significantly increased the effect of gefitinib (IC50 = 5.9 μM) and erlotinib (IC50 = 1.7 μM) at all concentrations. Similar results were obtained with cetuximab. Comparing the experimental and Bliss theoretical curves, the effect of the combination treatments was synergistic in the SUM-1315 cell line. These data revealed that EGFR inhibition using mAbs was active only in MDA-MB-468 and SUM-1315 cells, whereas all cell lines were sensitive to both erlotinib and gefitinib. Dual targeting EGFR using both mAbs and EGFR-TKIs appeared to have synergistic effects only in mAb-sensitive TNBC cell lines. Under the same experimental conditions, we also performed a cell viability assay on SUM-1315 cells transfected with wild-type *BRCA1* to determine whether BRCA1 deficiency increases sensitivity to anti-EGFR drugs. The results from these experiments demonstrated that reintroduction of full-length *BRCA1* did not reverse sensitivity of SUM-1315 cells to anti-EGFR drugs. The data from the viability assays on SUM-1315 and SUM1315-BRCA1 were similar (data not shown).

### Inhibition of RAS/MAPK pathway sensitizes TNBC cell lines to EGFR inhibitors

**Figure 3:**
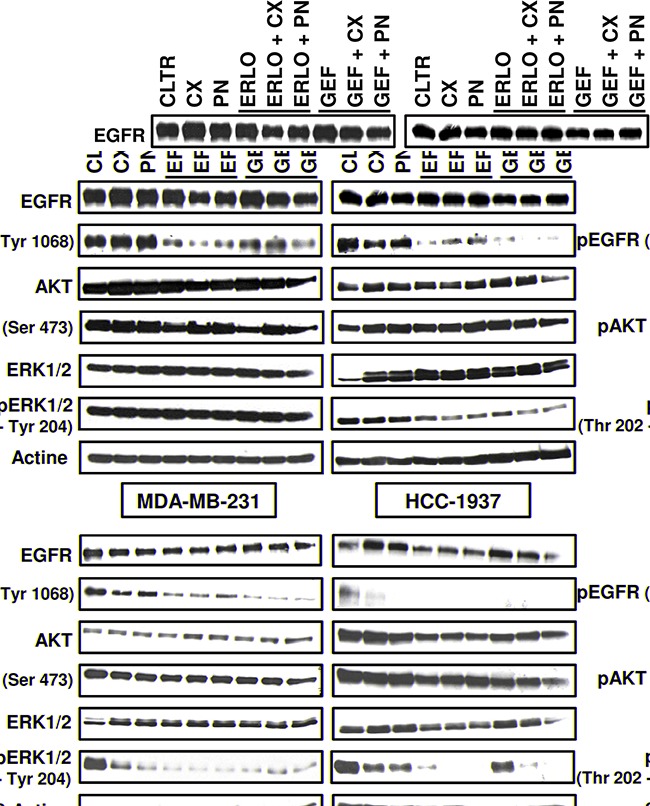
Western blot analysis of EGFR, AKT, ERK 1/2 and their phosphorylated forms in TNBC cell lines treated with a combination of anti-EGFR-targeted therapies Cell lines were exposed to 5 μM of erlotinib (ERLO) or gefitinib (GEF) and 10 μg/ml of cetuximab (CX) or panitumumab (PN) for 24 h. Fifty μg of whole cell protein extract was analyzed via 10% SDS-PAGE and immunoblotted using the indicated antibodies. Beta-actin antibody was used as a loading control. The data shown are representative of two independent experiments.

We next investigated the effects of mAbs and EGFR-TKIs on activation status of the RAS/MAPK and PI3K/AKT signaling pathways (Figure 3). Cells were treated for 24 h with fixed concentrations of EGFR-TKIs (5 μM) and mAbs (10 μg/ml) as single agents or in combination. Gefitinib and erlotinib were used at a concentration of 5 μM because this concentration is close to the median peak plasma concentrations reported in clinical pharmacokinetics studies [29, 30]. Resistance to anti-EGFR mAbs was associated with no effect of mAbs on EGFR pathway activation in MDA-MB-231 and HCC-1937 cells. As expected, cetuximab and panitumumab did not inhibit phosphorylation of AKT or phosphorylation of ERK1/2 in either cell line. By contrast, addition of EGFR-TKIs clearly inhibited EGFR phosphorylation in both cell lines. In MDA-MB-231 cells, erlotinib and gefitinib exposure reduced EGFR phosphorylation by up to 5-fold and 2.5-fold, respectively. In HCC-1937 cells, EGFR-TKIs decreased EGFR phosphorylation by up to 10-fold compared with untreated cells. Nevertheless, no significant effect was observed on total or activated forms of AKT. In the MDA-MB-231 cell line, anti-EGFR therapies had no impact on the expression or activation of ERK1/2. In the HCC-1937 cell line, all compounds tested enhanced the expression of ERK1, and only the addition of EGFR-TKIs significantly inhibited ERK1/2 phosphorylation (up to 5-fold decrease).

Regarding the sensitive MDA-MB-468, phosphorylation of EGFR was markedly reduced only in the presence of EGFR-TKIs (3.5-fold and 10-fold, respectively after erlotinib and gefitinib exposure). In SUM-1315 cells, all treatments completely suppressed the signal of EGFR phosphorylation. In these two cell lines, therapies had no significant effects on PI3K/AKT pathway as amounts of total and phosphorylated forms of AKT appeared not to differ compared with untreated cells. By contrast, both mAbs and EGFR-TKIs blocked the RAS/MAPK signaling pathway by inhibiting the phosphorylation of ERK1/2. In MDA-MB-468 cells, phospho-ERK1/2 was down-regulated by 2.5-fold and 5-fold, respectively, after cetuximab and panitumumab treatments. EGFR-TKIs were able to reduce the phosphorylation of ERK1/2 up to 10-fold compared with untreated cells. In SUM-1315 cells, mAbs and gefitinib as single agents blocked the phosphorylation of ERK1/2 less effectively than erlotinib. Cetuximab and panitumumab reduced phospho-ERK1/2 by 2-fold and 2.5-fold, respectively. Gefitinib and erlotinib decreased ERK1/2 phosphorylation by 2-fold and 10-fold, respectively, in comparison with untreated cells. The combination of gefitinib with either cetuximab or panitumumab completely suppressed ERK1/2 phosphorylation. Overall, the differential impact of anti-EGFR therapies observed on cell viability was associated with the effect on activation status of the RAS/MAPK pathway. These results suggest that inhibiting EGFR phosphorylation and reducing ERK1/2 activity sensitize TNBC cell lines to anti-EGFR-targeted therapies.

### Anti-EGFR sensitivity of TNBC cell lines is associated with cell cycle arrest at the G1 phase followed by apoptosis

**Figure 4:**
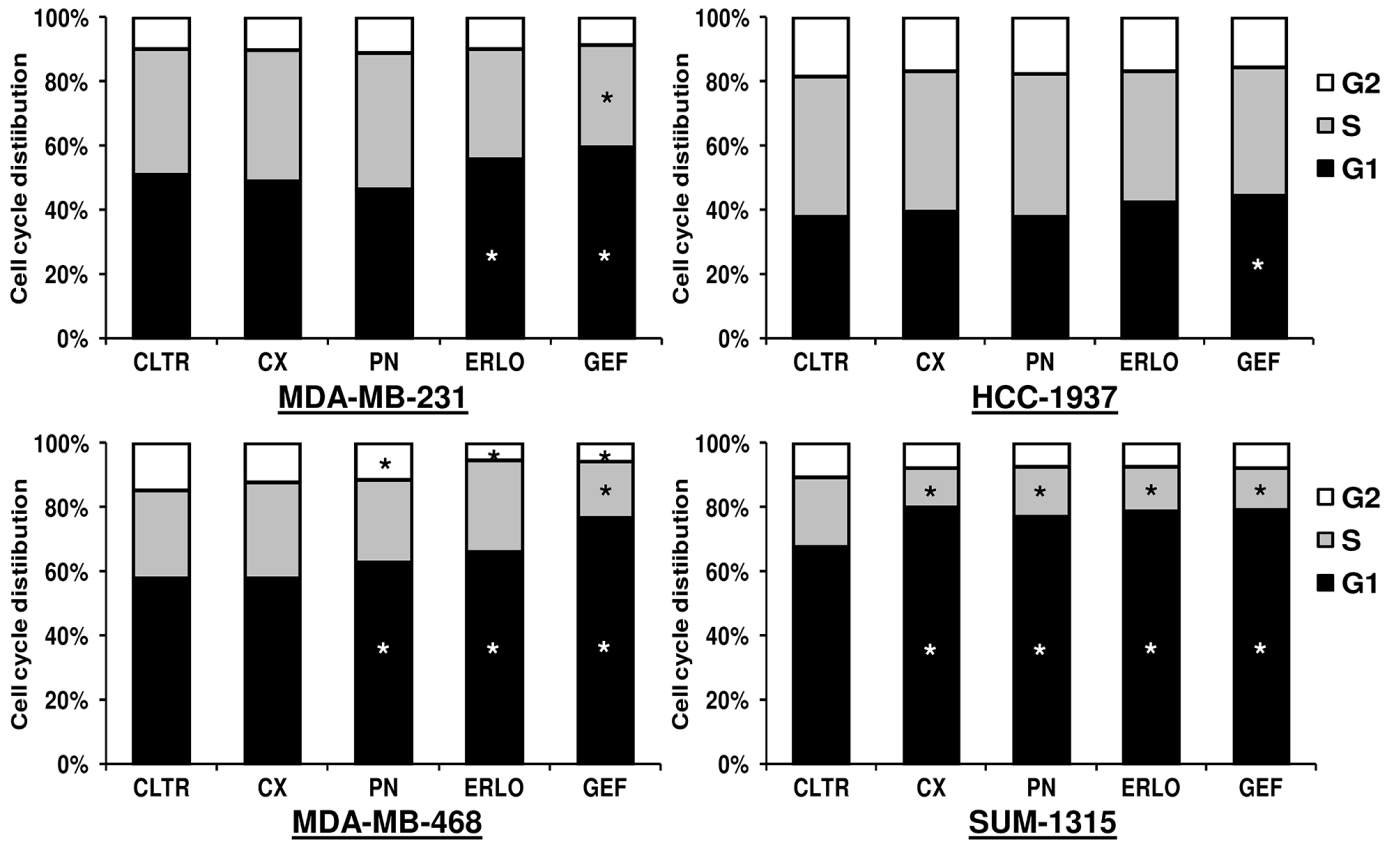
Effect of anti-EGFR-targeted therapies on cell cycle progression in TNBC cell lines Cells were treated for 48 h with 10 μg/ml of cetuximab (CX) or panitumumab (PN) and 5 μM of erlotinib (ERLO) or gefitinib (GEF) and stained with propidium iodide. Fluorescence was analyzed for cell cycle distribution by flow cytometry. The results are the mean values of three independent experiments. *p<0.05 for comparison between cells treated and cells untreated (CLTR) using Student's t-test.

**Figure 5:**
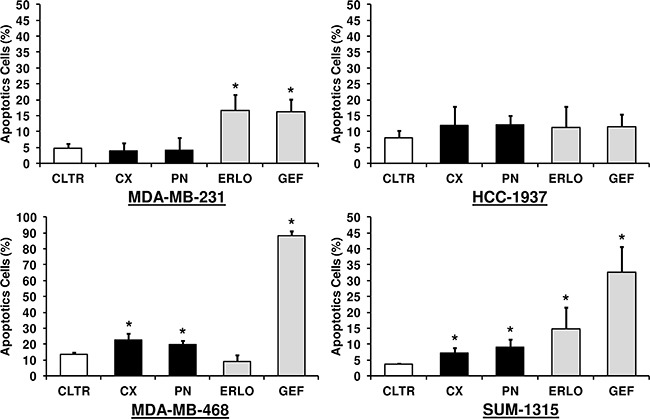
Apoptotic effect of anti-EGFR-targeted therapies in TNBC cell lines Cells were treated for 48 h with 10 μg/ml of cetuximab (CX) or panitumumab (PN) and 5 μM of erlotinib (ERLO) or gefitinib (GEF). Analysis of apoptosis was performed by both Annexin V-FITC and propidium iodide staining. Quantification of positive cells was evaluated by flow cytometry, and apoptotic cells were expressed as a percentage of total cell number. The data represent the mean values ± SEM of triplicate experiments. *p<0.05 for comparison between treated cells and untreated cells (CLTR) using Student's t-test.

We performed cell cycle and apoptosis analysis on TNBC cell lines in response to anti-EGFR-targeted therapies. The distribution of cell cycle phases and the proportion of apoptotic cells were determined after 48 h of treatment with 10 μg/ml of cetuximab or panitumumab and 5 μM of gefitinib or erlotinib. Combinations of EGFR-TKIs with mAbs were also tested (data not shown). As shown in Figure 4 and Figure 5, mAbs did not affect either cell cycle distribution or the proportion of apoptotic cells in MDA-MB-231 and HCC-1937 cell lines. Both EGFR-TKIs induced cell cycle arrest at the G1 phase (erlotinib p = 0.033; gefitinib p = 0.002) followed by apoptosis (erlotinib p = 0.034; gefitinib p = 0.032) in MDA-MB-231 cells. Gefitinib treatment also decreased the proportion of MDA-MB-231 cells in S phase. In HCC-1937 cells, neither drug had an effect on apoptosis compared with untreated cells. However, only gefitinib significantly inhibited cell cycle progression in HCC-1937 cells (p = 0.042). In the MDA-MB-468 cell line, panitumumab (p = 0.025) and both gefitinib (p = 0.005) and erlotinib (p = 0.006) caused significant G1 cell cycle arrest compared with untreated cells. The apoptotic rate markedly increased from 14% to 88% by gefitinib treatment, whereas erlotinib appeared to have no effect on cell death compared with untreated cells. Both mAbs were also able to induce apoptosis in the MDA-MB-468 cell line. Concerning the SUM-1315 cell line, the proportion of cells in the G1 phase markedly increased in response to both antibodies (cetuximab p = 0.003; panitumumab p = 0.011) and EGFR-TKIs (erlotinib p = 0.005; gefitinib p = 0.001) treatments compared with untreated cells. All treatments also significantly increased apoptosis in SUM-1315 cells. Treatments with cetuximab and panitumumab induced a 2 and 2.5-fold increase of apoptosis, respectively, compared with untreated SUM-1315 cells. Both erlotinib and gefitinib exposure also induced marked apoptosis (4 and 8.5-fold increase, respectively). In all these cell lines, combinatorial treatments (EGFR-TKIs and mAbs) did not significantly inhibit cell cycle or induce apoptosis compared with cells treated with single agents (data not shown). These results suggest that an anti-proliferative effect was due, at least in part, on induction of cell cycle arrest at G1 phase and apoptosis.

### Sensitivity to EGFR inhibitors reveals overexpression of genes involved in EGFR signaling pathways and down-expression of cyclin genes

We analyzed the expression of 43 genes involved in cell cycle control, apoptosis, angiogenesis, DNA repair and drug resistance. Some of these genes also encode signaling proteins and receptor tyrosine kinases (Table 2). The expression of these genes was assessed in cells exposed to EGFR-TKIs and mAbs as single agents and in combination for 48 h.

**Table 2:** List of selected gene expression assays used for configuring the Taqman low-density array cards

Gene symbol	Assay reference	Gene name
***Endogenous genes***		
*18S*	Hs99999901_s1	-
*GAPDH*	Hs99999905_m1	glyceraldehyde-3-phosphate dehydrogenase
***PI3K/AKT and RAS/MAPK signaling pathways***		
*EGFR*	Hs01076078_m1	epidermal growth factor receptor
*IGF1R*	Hs00609566_m1	insulin-like growth factor 1 receptor
*MET*	Hs00179845_m1	met proto-oncogene
*HER3*	Hs00176538_m1	human epidermal growth factor receptor 3
*PIK3CA*	Hs00180679_m1	phosphatidylinositol-4.5-bisphosphate 3-kinase. catalytic subunit alpha
*PTEN*	Hs00829813_s1	phosphatase and tensin homolog
*AKT1*	Hs00178289_m1	v-akt murine thymoma viral oncogene homolog 1
*MTOR*	Hs00234508_m1	mechanistic target of rapamycin (serine/threonine kinase)
*BRAF*	Hs00269944_m1	v-raf murine sarcoma viral oncogene homolog B
*KRAS*	Hs00364282_m1	Kirsten rat sarcoma viral oncogene homolog
*MAPK1*	Hs01046830_m1	mitogen-activated protein kinase 1
*MAPK3*	Hs00385075_m1	mitogen-activated protein kinase 3
*FOS*	Hs00170630_m1	FBJ murine osteosarcoma viral oncogene homolog
*MYC*	Hs00153408_m1	v-myc avian myelocytomatosis viral oncogene homolog
***Cell cycle control***		
*E2F1*	Hs00153451_m1	E2F transcription factor 1
*CDK2*	Hs01548894_m1	cyclin-dependent kinase 2
*CDK4*	Hs00175935_m1	cyclin-dependent kinase 4
*CDK6*	Hs01026372_m1	cyclin-dependent kinase 6
*CDKN1A*	Hs00355782_m1	cyclin-dependent kinase inhibitor 1A
*CDKN2A*	Hs00923893_m1	cyclin-dependent kinase inhibitor 2A
*CDKN1B*	Hs00153277_m1	cyclin-dependent kinase inhibitor 1B
*CDKN1C*	Hs00175938_m1	cyclin-dependent kinase inhibitor 1C
*CCNA1*	Hs00171105_m1	cyclin A1
*CCNB1*	Hs99999188_m1	cyclin B1
*CCND1*	Hs00277039_m1	cyclin D1
*CCNE1*	Hs00233356_m1	cyclin E1
*CHEK1*	Hs00967506_m1	checkpoint kinase 1
*CHEK2*	Hs00200485_m1	checkpoint kinase 2
*RB1*	Hs01078066_m1	retinoblastoma 1
*TP53*	Hs99999147_m1	tumor protein p53
***Apoptosis***		
*BAX*	Hs00180269_m1	BCL2-associated X protein
*BCL2*	Hs00608023_m1	B-cell CLL/lymphoma 2
*CASP8*	Hs01018151_m1	caspase 8
*CASP9*	Hs00154260_m1	caspase 9
*CASP3*	Hs00234387_m1	caspase 3
*XIAP*	Hs00745222_s1	X-linked inhibitor of apoptosis
***Angiogenesis***		
*NOS2*	Hs01075529_m1	nitric oxide synthase 2. inducible
*CDH1*	Hs01023894_m1	cadherin 1, type 1, E-cadherin (epithelial)
*PLAU*	Hs01547054_m1	plasminogen activator. urokinase
*VEGF*	Hs00900055_m1	vascular endothelial growth factor A
*MMP9*	Hs00234579_m1	matrix metallopeptidase 9
***DNA repair***		
*BRCA1*	Hs00173237_m1	breast cancer 1
*PARP1*	Hs00242302_m1	poly (ADP-ribose) polymerase 1
*PARP2*	Hs00193931_m1	poly (ADP-ribose) polymerase 2
***Drug resistance***		
*ABCB1*	Hs00184491_m1	ATP-binding cassette, sub-family B (MDR/TAP), member 1
*ABCG2*	Hs00184979_m1	ATP-binding cassette, sub-family G (BCRP1), member 2

**Figure 6:**
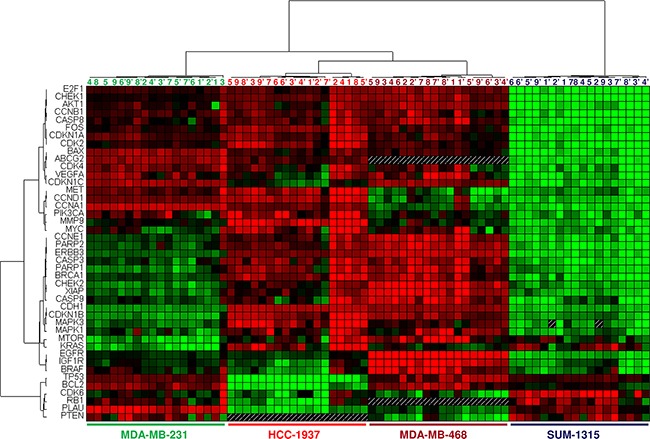
Profiles of differentially expressed genes in TNBC cell lines used in this study The data are presented in heat map format combined to hierarchical clustering. Each row represents a gene, and each column represents a cell line and treatments. The expression of each gene is relative to the mean gene expression in all cell lines and is illustrated according to a color scale from green to red. Genes in red and green indicate expression above and below the median, respectively. Cell lines were exposed for 48 h to 5 μM of erlotinib (erlo) or gefitinib (gef) and/or 10 μg/ml of cetuximab (cx) or panitumumab (pn). Two independent experiments were performed; 1-1′: untreated cells; 2-2′: cx; 3-3′: pn; 4-4′: erlo; 5-5′: erlo + cx; 6-6′: erlo + pn; 7-7′: gef; 8-8′: gef + cx; 9-9′: gef + pn.

The results are presented in heat map format combined with hierarchical clustering, which allows for the distribution of genes according to their expression in each experimental condition (Figure 6). Hierarchical cluster analysis grouped each cell line into pure clusters, regardless of treatments. The data produced a dendrogram, with cell lines falling into two groups characterized by different gene expression profiles. Overall, gene expression was lower in SUM-1315 cells than in the others. A cluster of 37 down-regulated genes clearly discriminated this cell line from others. This cluster included 12 genes involved in cell cycle control (*E2F1*, *CDK2*, *CDK4*, *CDKN1A*, *CDKN1B*, *CDKN1C*, *CCNA1*, *CCNB1*, *CCND1*, *CCNE1*, *CHEK1* and *CHEK2*) and 5 genes involved in apoptosis (*BAX, CASP8, CASP9, CASP3* and *XIAP*). In the cluster tree of cell lines, HCC-1937 and MDA-MB-468 were the closest, whereas MDA-MB-231 and SUM-1315 were the most distant. Typology of these dendrograms was consistent with results from cytotoxicity and cell cycle experiments, suggesting a correlation between gene expression profile and sensitivity of TNBC cell lines to EGFR inhibitors.

To evaluate the effect of EGFR inhibitors on gene expression, relative quantification (RQ) of each gene was calculated. Fold changes were calculated relative to the RQ value of untreated cells, which was equal to one. As no significant difference was observed between cetuximab and panitumumab treatments, as well as between gefitinib and erlotinib treatments, we calculated the average RQ value of each gene for EGFR-TKI and mAb treatments. Genes were considered differentially expressed if their RQ was > 1.5-fold up- or down-regulated compared with untreated cells (RQ value > 1.5 or RQ value < 0.6, respectively). Relative quantification values of these genes are summarized in Table 3. By these criteria, four genes of signaling pathways (*EGFR*, *PIK3CA*, *AKT1* and *MAPK1*) were up-regulated by anti-EGFR therapies in the MDA-MB-231 cell line compared with untreated cells. No gene expression changes related to cell cycle regulation and apoptosis were observed in this cell line. Following treatment of HCC-1937 cells, the expression of four genes was significantly decreased, among which three genes encode for cyclins (*CCNA1*, *CCND1* and *CCNE1*) and one is involved in cell migration and angiogenesis (*MMP9*). Two others genes, *MAPK3* and *ABCG2*, were up-regulated by combination of EGFR inhibitors (respectively up to 1.7 and 1.5-fold). Interestingly, increased expression of the drug resistance gene *ABCG2*, induced by the combination of treatments was statistically significant compared with EGFR-TKIs alone (p = 0.032).

**Table 3:** Relative quantification (RQ) of differentially expressed genes using biological significance (> or < 1.5-fold: RQ > 1.5 or RQ < 0.6 respectively)

MDA-MB-231				HCC-1937			
Genes	mAbs	TKIs	mAbs + TKIs	Genes	mAbs	TKIs	mAbs + TKIs
*Signaling pathways*				*Signaling pathways*			
***EGFR***	1.7 ± 0.7	1.4 ± 0.3	1.8 ± 0.3	***MAPK3***	1.6 ± 1.0	1.3 ± 0.5	1.7 ± 1.4
***PIK3CA***	1.5 ± 0.6	1.8 ± 0.4	1.6 ± 0.5	*Cell cycle*			
***AKT1***	2.2 ± 1.4	2.1 ± 1.3	2.3 ± 1.1	***CCNA1***	0.7 ± 0.1	0.6 ± 0.3	0.6 ± 0.2
***MAPK1***	1.6 ± 0.5	1.7 ± 0.3	1.7 ± 0.7	***CCND1***	0.8 ± 0.2	0.6 ± 0.2	0.7 ± 0.2
				***CCNE1***	0.8 ± 0.2	0.6 ± 0.2	0.7 ± 0.3
**MDA-MB-468**				*Angiogenesis*			
**Genes**	**mAbs**	**TKIs**	**mAbs + TKIs**	***MMP9***	0.4 ± 0.2	0.4 ± 0.1	0.4 ± 0.1
*Signaling pathways*				*Drug resistance*			
***MTOR***	0.4 ± 0.1	0.6 ± 0.	0.5 ± 0.1	ABCG2*	**0.8 ± 0.2**	**1.0 ± 0.4**	**1.5 ± 0.5**
***MAPK1***	1.1 ± 0.2	1.4 ± 0.7	1.5 ± 1.1				
***MAPK3***	1.7 ± 0.1	2.2 ± 0.1	1.7 ± 0.1				
***MYC***	0.5 ± 0.0	0.4 ± 0.1	0.4 ± 0.1	**SUM-1315**			
*Cell cycle*				**Genes**	**mAbs**	**TKIs**	**mAbs + TKIs**
***CCNA1***	0.1 ± 0.1	0.1 ± 0.0	0.1 ± 0.0	*Signaling pathways*			
***CCNB1*** *	**1.0 ± 0.3**	**0.7 ± 0.1**	**0.5 ± 0.1**	***PIK3CA***	1.5 ± 0.8	1.6 ± 1.3	1.6 ± 1.1
***CCND1*** *	**0.3 ± 0.1**	**0.1 ± 0.1**	**0.1 ± 0.0**	***PTEN***	2.4 ± 1.3	1.2 ± 0.4	1.7 ± 0.2
***CCNE1*** *	**0.7 ± 0.1**	**0.7 ± 0.1**	**0.5 ± 0.1**	***MAPK1***	2.1 ± 1.5	2.0 ± 1.0	2.0 ± 1.1
***CHEK1***	0.7 ± 0.1	0.7 ± 0.1	0.6 ± 0.1	***MAPK3***	1.1 ± 0.9	1.7 ± 1.2	1.4 ± 0.6
*Apoptosis*				*Cell cycle*			
***BCL2***	0.7 ± 0.1	0.8 ± 0.2	0.6 ± 0.1	***E2F1***	0.5 ± 0.2	0.6 ± 0.1	0.5 ± 0.1
***CASP8***	0.6 ± 0.2	0.6 ± 0.2	0.5 ± 0.1	***CDK2***	0.5 ± 0.1	0.6 ± 0.1	0.5 ± 0.1
***XIAP*** *	**0.7 ± 0.2**	**0.7 ± 0.1**	**0.5 ± 0.1**	***CDKN1C*** *	**2.9 ± 1.0**	**3.1 ± 0.4**	**4.4 ± 1.2**
*Angiogenesis*				***CCNB1***	0.6 ± 0.1	0.6 ± 0.1	0.5 ± 0.1
***MMP9***	1.3 ± 0.8	0.6 ± 0.1	0.5 ± 0.2	***CCND1***	0.6 ± 0.2	0.7 ± 0.2	0.6 ± 0.1
*DNA repair*				*Drug resistance*			
***PARP1***	0.6 ± 0.2	0.5 ± 0.1	0.5 ± 0.1	***ABCB1***	0.7 ± 0.4	2.4 ± 0.8	1.5 ± 0.7

mAbs: Average of relative quantification of gene expression after cetuximab and panitumumab treatments (10 μg/ml). TKIs: Average of relative quantification of gene expression after gefitinib and erlotinib treatments (5 μM).

* p < 0.05: Genes with statistically significant difference between monotherapies and combination using Student's t-tests.

In MDA-MB-468, 14 genes were differentially expressed upon anti-EGFR treatment. Among the genes of signaling pathways, *MAPK1* and *MAPK3* were overexpressed, with 1.5 and 2.2-fold up-regulation, respectively. Genes of MTOR kinase and MYC transcription factor were approximately 2-fold down-regulated in MDA-MB-468 treated cells compared with untreated cells. All other genes were also markedly down-regulated, among which five genes are involved in cell cycle progression (*CCNA1*, *CCNB1*, *CCND1*, *CCNE1*, *CHEK1*), and three genes belong to the apoptosis signaling cascade (*BCL2*, *CASP8* and *XIAP*). The expression of three cyclin genes (*CCNB1*, *CCND1* and *CCNE1*) and an anti-apoptotic gene (*XIAP*) was statistically down-regulated upon combination treatment compared with gefitinib or erlotinib alone. Treatments with EGFR-TKIs alone or in combination with mAbs also induced down-regulation of *MMP9* and *PARP1* genes in this cell line.

The quantification of gene expression in the SUM-1315 cell line revealed significant modifications in 10 genes after anti-EGFR treatment. Four genes belonging to EGFR signaling pathways were overexpressed: *PIK3CA*, *PTEN*, *MAPK1* and *MAPK3*. Among the genes of cell cycle regulation, *E2F1*, *CDK2*, *CCNB1* and *CCND1* genes were down-regulated, and the cyclin-dependent kinase inhibitor *CDKN1C* gene was markedly up-regulated following both types of EGFR inhibitor treatment (mAbs: 2.9-fold, EGFR-TKIs: 3.1-fold, combination: 4.4-fold). We also found that the drug resistance gene *ABCB1* was up-regulated in this cell line after exposure to EGFR-TKIs alone or in combination with mAbs (EGFR-TKIs: 2.4-fold, combination: 1.5-fold). Overall, the response to anti-EGFR therapies is characterized by overexpression of several genes encoding proteins involved in EGFR signaling pathways and down-regulation of cyclin genes.

## DISCUSSION

This study focused on the effect of the main anti-EGFR therapies currently approved for use in oncology on four TNBC cell lines that harbor different EGFR pathway mutations. A gene mutation analysis in breast cancer cell lines has reported genetic alterations of several key players in PI3K/AKT and RAS/MAPK pathways that induce their activation [31]. HCC-1937 and MDA-MB-468 cell lines have an acquired homozygous deletion of *PTEN*. The PTEN protein is a lipid phosphatase that blocks PI3K and negatively regulates the PI3K/AKT pathway. MDA-MB-468 also carries *EGFR* amplifications that are responsible for large amounts of EGFR protein. MDA-MB-231 cells harbor activated mutations of *KRAS* and *BRAF* that induce constitutive activation of ERK1/2. Hollestelle *et al.* demonstrated that, among 40 human breast cancer cell lines, only one shows mutational activation of both PI3K/AKT and RAS/MAPK signaling pathways, suggesting that mutations of these pathways in breast cancer are mutually exclusive [31].

The synergism of the combination of EGFR-TKIs and mAbs was limited to MDA-MB-468 and SUM-1315 cell lines. The MDA-MB-468 cell line had the highest amount of EGFR due to its *EGFR* amplification, and the SUM-1315 cell line expressed minimal EGFR levels. The response of cells to mAbs did not appear to be related to basal levels of EGFR and its phosphorylated forms. Previous studies in EGFR-expressing NSCLC patients as well as several cell lines have shown that sensitivity to cetuximab and both EGFR-TKIs does not correlate with EGFR expression levels [32–34]. Other studies on mCRC have demonstrated that the likelihood of response to either cetuximab or panitumumab is not associated with EGFR expression in tumors [35–37].

Overall, the growth-inhibitory effects of anti-EGFR therapies on TNBC cell lines were consistent with cell cycle and apoptotic profiles after treatment. Sensitivity to EGFR inhibitors was a consequence of cell cycle arrest at the G1 phase followed by apoptosis. Treatment with gefitinib alone led to stronger inhibitory effects on cell cycle and higher levels of apoptosis than others treatments. These results suggest that a combination of gefitinib and anti-EGFR mAbs should be the most appropriate strategy for the dual targeting of EGFR in TNBC. In the SUM-1315 cell line, both EGFR-TKIs and mAbs as single agents inhibited cell cycle and promoted apoptosis. Targeting EGFR has already been associated with cell cycle arrest at the G1 phase in several cancer cell lines and human tumor xenografts studies [38]. Cell cycle arrest of cancer cells led to apoptosis by altering the expression of multiple genes involved in the control of cell death [39, 40].

This study also showed a link between sensitivity to EGFR inhibitors and ERK inhibition. Cetuximab and panitumumab failed to inhibit the RAS/MAPK pathway in resistant cell lines (MDA-MB-231 and HCC-1937), whereas they reduced EGFR and ERK phosphorylation in sensitive cell lines (MDA-MB-468 and SUM-1315). In MDA-MB-231 cells, activated *RAS* and *RAF* mutants induced hyperphosphorylation of ERK1/2 proteins and prevented their inactivation by mAbs. As breast cancers and colorectal cancers have similar *BRAF* and *KRAS* mutation spectra, it is logical that both cancers have a similar response to both cetuximab and panitumumab. The relationship between responses to anti-EGFR mAbs and *KRAS*-mutation status in mCRC is well supported [41, 42]. The observations of the effect of cetuximab and panitumumab on the HCC-1937 cell line suggested that mutational activation of the PI3K/AKT pathway through *PTEN* deletion may be involved in resistance to mAbs. The PI3K/AKT signaling pathway has been reported to cross-talk with the RAS/MAPK pathway through RAS activation [43]. Thus, loss of *PTEN* leads to constitutive activation of the PI3K/AKT pathway and could induce RAS/MAPK signaling downstream. Moreover, it is conceivable that the induction of ERK1 (mRNA and protein) observed upon mAb treatment also contributes to resistance of the HCC-1937 cell line.

The data from HCC-1937 and MDA-MB-468 cell lines were different, though they both had *PTEN* homo deletions. Loss of *PTEN* expression induced activation of the PI3K/AKT pathway, resulting in increased phosphorylation of AKT in both cell lines. Several studies have shown that loss of *PTEN* is associated with resistance to anti-EGFR mAbs in EGFR-expressing mCRC [31, 34, 44, 45]. In the MDA-MB-468 cell line, mutational activation of the PI3K/AKT pathway through *PTEN* deletion should be involved in resistance to mAbs; however, we observed that cells could have positive responses to both mAbs. Clinical studies have reported that an increased *EGFR* gene copy number is predictive of clinical responsiveness to EGFR targeted therapies in mCRC patients [46, 47]. These data provide evidence that *EGFR* amplification could be more predictive of the response to anti-EGFR mAbs in TNBC than *PTEN* status if tumors harbor both genetic alterations. Moreover, *EGFR* amplification was found in a large proportion of TNBC patients, ranging from 30 to 85% according [48–50].

The SUM-1315 cell line has no mutational activation of EGFR signaling pathways; however, like HCC-1937, it carries homozygous deleterious mutations in *BRCA1* [51]. These BRCA1 mutations render the BRCA1 protein inactive or nonfunctional, and cells lose their ability to repair DNA efficiently. It has been demonstrated that none of these BRCA1 mutants expresses nuclear BRCA1 in these two cell lines [51]. Furthermore, data from viability assays have shown that reintroduction of *BRCA1* in SUM-1315 cells does not reverse sensitivity to anti-EGFR drugs. In this cell line, all treatments impaired kinase activity of EGFR, and combined treatments synergistically reduced levels of phospho-ERK1/2, supporting the concept that ERK1/2 inactivation plays a central role in predicting response to EGFR inhibitors. Baselga et al. already investigated the downstream signaling pathways of EGFR and suggest that down-regulated activity of ERK1/2 after treatment with anti-EGFR drugs might serve as a marker of drug response [19, 52]. Indeed, mAbs and EGFR-TKIs did not induce discernible changes in phospho-AKT levels in all cell lines tested. These results are in agreement with previous reports demonstrating that inhibition of EGFR tyrosine kinase activity reduces ERK1/2 but not AKT phosphorylation in tumors from breast cancer patients [19]. There is strong evidence that the RAS/MAPK signaling pathway is involved in the promotion of cell proliferation and the prevention of apoptosis [53]. Preventing activation of ERK1/2 inhibits cell proliferation by blocking G1-phase progression [54]. Activated ERK1/2 induces phosphorylation of transcription factors which bind the promoter regions of targeted genes involved in cell cycle, cell death and drug resistance [53]. Constitutive activation of ERK1/2 proteins increases the transcription of cyclins and the expression of cyclin-dependent kinase inhibitors (CDKIs), which arrest cell cycle at the G1 phase in response to DNA damage. Moreover, the RAS/MAPK pathway increases the expression of drug pumps and anti-apoptotic molecules [53, 55]. Transcriptional analysis of genes selected in this study revealed that inhibition of ERK1/2 activation in sensitive cells was associated with the induction of positive regulators of cell cycle and inactivation of negative regulators. Among the genes differentially expressed in MDA-MB-468 cells, five genes involved in cell cycle progression (*CCNA1*, *CCNB1*, *CCND1*, *CCNE1*, *CHEK1*) were down-regulated by anti-EGFR therapies. We also demonstrated that the combination of mAbs and EGFR-TKIs significantly reduced anti-apoptotic *XIAP* and *BCL2* genes in the MDA-MB-468 susceptible cell lines. It has been reported that *XIAP* and *BCL2* confer acquired resistance to gefitinib in TNBC cell lines [56]. It has also been demonstrated in non-small cell lung cancer cell lines (NSCLC) that EGFR-TKIs exert their cytotoxic effects through inhibition of the apoptosis-related protein BCL2 [57]. In the SUM-1315 cell line, treatments inhibited two cyclins, CCNB1 and CCND1, which are involved in the G1 to S phase transition. We also found that CDK2 was inhibited by treatments. An in vitro study using several breast cancer cell lines has shown that down-regulation of CDK2 after treatment with erlotinib was correlated with a reduction of cell viability. The authors found that blocking CDK2 activity led to increased sensitivity to erlotinib in EGFR-expressing TNBC cell line [58]. The E2F1 transcription factor was also down-regulated in SUM-1315 cells. E2F1 is known to induce the transcription of genes required for the G1/S transition [59]. Moreover, combined treatments induced synergistic effects on RAS/MAPK and elicited cell cycle arrest by enhancing the transcription of CDKN1C. CDKN1C is known to suppress E2F1 transcriptional activity. Synergistic growth-inhibitory effects induced by combined treatment in the SUM-1315 cell line may, in part, be caused by inactivation of *E2F1* [60, 61]. Moreover, the anti-proliferative effect of gefitinib has been reported to be associated with suppression of E2F1 expression in several cancer cell lines [62].

**Figure 7:**
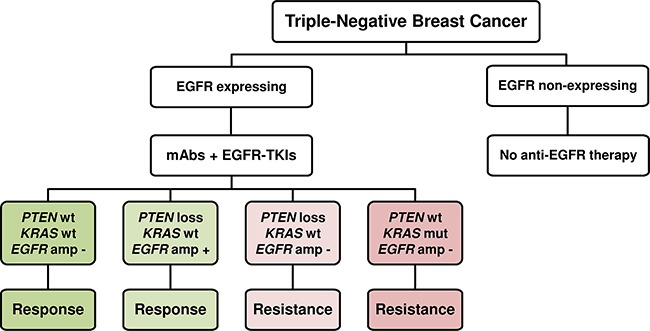
A suggested algorithm for predicting the response to treatment with a combination of dual EGFR inhibitory agents according to *EGFR*, *KRAS* and *PTEN* status mAbs: monoclonal antibodies; EGFR-TKIs: tyrosine kinase inhibitors; wt: wild-type; mut: mutated; amp: amplification.

In conclusion, we have demonstrated that dual targeting of EGFR with a combination of mAbs and EGFR-TKIs induces synergistic effect in mAb-sensitive cell lines. Several lines of evidence showed that *EGFR*, *KRAS* and *PTEN* status may predict the response to mAbs in TNBC. These results could allow proposal of an algorithm for the selection of TNBC patients suitable for a combination of anti-EGFR-targeted therapies (Figure 7). The detection of EGFR expression in neoplastic tissues is required to identify EGFR-expressing TNBC. For non-expressing EGFR TNBC, a combination of anti-EGFR therapy may not be efficient. Although it has been demonstrated that the response of cells to mAbs is not related to basal levels of EGFR, clinical studies have already confirmed that EGFR expression is necessary for the anti-EGFR mAbs to be active [34–36]. Moreover, both cetuximab and panitumumab are approved for the treatment of patients with EGFR-expressing metastatic colorectal cancer [63, 64]. For EGFR-expressing TNBC, we suggest an algorithm of molecular diagnostics to predict the clinical response to this therapeutic approach. It is of interest to investigate the mutational status of *KRAS*, *EGFR* and *PTEN* for the evaluation of EGFR inhibitors. Several studies have indicated that *KRAS* mutations are rare in triple-negative breast tumors, supporting the use of anti-EGFR-targeted therapies for their treatment [65, 66]. However, the loss of *PTEN* and *EGFR* amplification occurs more frequently. Loss of PTEN was found to be mutated at 35% incidence and *EGFR* was found to be amplified at 30 to 85% incidence in TNBC [48–50, 67–70]. A screening of TNBC tumors for status of these three genes could help identify patients who may benefit from this therapeutic strategy. In particular, patients with *EGFR* amplification and/or wild-type *KRAS* and *PTEN* should be considered for anti-EGFR mAbs treatment combined with EGFR-TKIs. These results need to be validated in a large prospective clinical trial. Even so, this study provides additional preclinical evidence that the mutation status of EGFR signaling pathways in TNBC should be considered for use of combined treatment with dual EGFR inhibitors.

## MATERIALS AND METHODS

### Cell lines, culture conditions and drug preparation

The cell lines used in this study were conserved in the Biological Resource Center of Jean Perrin Comprehensive Cancer Center, identified under No. BB-0033-00075 (Clermont-Ferrand, France). MDA-MB-231 (HTB-26), MDA-MB-468 (HTB-132), HCC-1937 (CRL-2336) and MCF-7 (HTB-22) breast cancer cell lines were obtained from the American Type Culture Collection (ATCC; Manassas, VA, USA), and the SUM-1315 (SUM1315M02) cell line was purchased from Asterand (Detroit, MI, USA). All these cell lines are classified as basal-like TNBC cell lines except MCF-7, which is a non-TNBC cell line. As shown in Table 4, some of these cell lines harbor mutations in EGFR pathway genes [71–73]. SUM-1315 has no mutational activation of EGFR signaling pathways. This allowed us to investigate the effect of drugs according to the presence or absence of mutations that could affect the therapeutic response to anti-EGFR drugs. SUM-1315 and HCC-1937 are two BRCA1-defective TNBC cell lines harboring, the 185delAG and 5382insC BRCA1 mutations, respectively. According to the Breast Cancer Information Core (BIC) mutation database (http://research.nhgri.nih.gov/bic/), the BRCA1 mutations found in SUM-1315 and HCC-1937 cell lines have been classified as pathogenic mutations and are similar to those frequently found in BRCA1 mutant breast cancer families [51, 74].

**Table 4:** Characteristics of triple-negative breast cancer cell lines used in this study

Cell lines	Basal subtype	Histology	Mutations in EGFR pathways	Other mutations
**MDA-MB-231**	Basal-like B	IDC	*BRAF, KRAS*	*CDKN2A, PDGFR, TP53*
**HCC-1937**	Basal-like A	DC	*PTEN*	*BRCA1, MDC1, TP53*
**MDA-MB-468**	Basal-like A	DC	*PTEN, EGFR amplification*	*RB1, SMAD4, TP53*
**SUM-1315**	Basal-like B	DC	-	*BRCA1, TP53, CDKN2A*

Data were adapted from [71–73] and COSMIC database (http://www.sanger.ac.uk/genetics/CGP/CellLines/). DC: ductal carcinoma; IDC: invasive ductal carcinoma.

Cells were maintained in monolayer cultures at 37°C in a humidified atmosphere of 95% air and 5% CO_2_, except for MDA-MB-231 and MDA-MB-468 cells, which were grown without CO_2_. MDA-MB-468 and MDA-MB-231 cells were cultured in L-15 medium, while HCC-1937 and MCF-7 cells were respectively grown in RPMI 1640 and DMEM medium (Invitrogen Life Technologies, Carlsbad, CA, USA). The media were supplemented with 10% heat-inactivated fetal bovine serum (FBS), 2 mM L-glutamine and 20 mg/mL gentamicin. SUM-1315 cells were cultured in Ham's F-12 medium supplemented with 5% FBS, 1% HEPES buffer, 10 ng/ml EGF and 5 μg/ml insulin (Invitrogen Life Technologies, Carlsbad, CA, USA). The two anti-EGFR monoclonal antibodies (mAbs) cetuximab (Erbitux®; Merck Pharma, Darmstadt, Germany) and panitumumab (Vectibix®; Amgen Inc, Thousand Oaks, CA, USA) were provided by the pharmacy of the Jean Perrin Comprehensive Cancer Center and stored at 4°C. The two selective EGFR-TKIs gefitinib (Iressa®; AstraZeneca, UK) and erlotinib (Tyverb®, Roche, Germany) were purchased from LC Laboratories (Woburn, MA, USA). EGFR-TKIs were dissolved in DMSO (stock solution at 40 μM), stored at −20°C and used within one month. Cells were treated with increasing concentrations of these drugs for 24 h or 48 h. The drugs were diluted immediately before use in growth medium. The final DMSO concentration always remained constant in all analyzed cell cultures, i.e., 0.2%.

### Cell viability assay

Cells were seeded into 96-well plates, and cell viability was assessed using the sulforhodamine B (SRB) assay (Sigma-Aldrich, St Louis, MO, USA) according to the method described by Vichai and Kirtikara [75]. Cells were seeded in sixplicate in 96-well plates and incubated overnight. Then, cells were treated with increasing concentrations of drugs for 24 h. The plates were washed twice with PBS and incubated for 72 h with complete cell culture media. The cells were fixed with trichloroacetic acid (final concentration 10%) at 4°C for 1 h. The plates were then washed 5 times with water, air dried, stained with 0.4% SRB dissolved in 1% acetic acid at room temperature for 30 min and subsequently washed 4 times with 1% acetic acid to remove unbound stain. A 10 mM Tris base solution was added to each well to solubilize the protein-bound dye. Viability was determined by measuring the optical density at 540 nm.

To determine whether BRCA1 deficiency increases the sensitivity of SUM-1315 cells to anti-EGFR drugs, we performed cell viability assays on SUM-1315 cells transfected with wild-type BRCA1. Two cell lines, SUM1315-BRCA1 and SUM1315-LXSN, were previously generated in our lab from the BRCA1 mutated SUM-1315 cell line [83]. The SUM1315-BRCA1 cell line was obtained by stable transfection of SUM-1315 cells with the BRCA1-encoding LXSN plasmid as previously described. It has been demonstrated that full-length BRCA1 transfection restores the expression of BRCA1 protein. SUM-1315-LXSN cells were obtained by stable transfection of SUM-1315 cells with an empty LXSN plasmid as a negative control [76].

Analysis of drug combination effects on cell viability was performed using the Bliss independence model, which allows for the calculation of the expected effect of combination therapy [77]. The inhibitory effects of drug combinations were expressed as the following equation: IAB = IA + IB – IA x IB, where IA and IB are the single agent inhibition levels at fixed concentrations. If the experimentally measured effect of the drug combination was equal to, higher than or lower than the expected effect (IAB), the combination was considered to be additive, synergistic or antagonistic, respectively.

### Western blotting

Cells were plated in 10 cm dishes at a density of 5 × 10^5^ cells per dish and treated with EGFR inhibitors the following day. After 24 h exposure to anti-EGFR therapies, cells were harvested, washed with PBS and lysed with lysis buffer containing RIPA buffer, 1% protease inhibitor and 1% phosphatase inhibitor cocktails (Sigma-Aldrich, St Louis, MO, USA). The lysate was kept on ice for 20 min, regularly vortexed and subsequently centrifuged at 12,000 g for 10 minutes at 4°C. The supernatant was collected, and protein concentrations were determined by the protein assay kit from Bio-Rad Laboratories (Hercules, CA, USA). Samples containing equal amounts of proteins (15 μg) were separated on 10% SDS-PAGE (Bio-Rad, Hercules, CA, USA) and transferred to PVDF membranes (GE healthcare, Westborough, MA, US). The membranes were blocked for 1 h with 5% milk powder in TBS-T buffer (1X Tris-buffered saline, 0.1% Tween) at room temperature then incubated overnight at 4°C with primary antibodies: anti-phospho-EGFR (Tyr 1068, Tyr 1045), anti-EGFR, anti-phospho-ERK1/2 (Thr 202, Tyr 204), anti-ERK1/2, anti-phospho-AKT (Ser 473), anti-AKT at final dilutions of 1:1000 (Cell Signaling Technology, Danvers, MA, USA) and anti-β-actin at a final dilution of 1:40,000 (Calbiochem, San Diego, CA, USA). After 3 washes with TBS-Tween, membranes were blotted for 1 h at room temperature with horseradish peroxidase-conjugated secondary antibodies: goat anti-rabbit IgG for selected proteins (1:2000 dilution; Cell Signaling Technology) and goat anti-mouse for β-actin (1:2000 dilution; Santa Cruz Biotechnology, Santa Cruz, CA, USA). After final washes, membranes were incubated in ECL Western Blotting Detection Reagent (Amersham Bioscience, Piscataway, NJ, USA), and detection was performed using an automated X-ray film processor (Hyperprocessor; GE healthcare, Westborough, MA, US). Quantification of Western blot signals (Figure 1 and Figure 3) was performed by computer-assisted densitometry using ImageJ software. The intensity of individual bands was expressed relative to β-actin, and the ratio of phosphoprotein/protein was determined. The fold changes in phosphorylation of EGFR, AKT and ERK1/2 between untreated cells and treated cells were also measured.

### Cell cycle analysis

Cells were seeded into 6-well plates with 5 x10^4^ cells per well. After overnight incubation, cells were treated or not with EGFR inhibitors for 48 h. Adherent cells were dissociated by trypsin and collected by centrifugation at 500 g for 10 min. Cell pellets were washed twice with PBS, and cell membranes were disrupted by repeated cycles of freezing and thawing in liquid nitrogen. Then, cells were incubated with 200 μl of ribonuclease A (1 mg/ml) and stained with 200 μl of propidium iodide solution (100 μg/ml) (Sigma-Aldrich, St Louis, MO, USA). Fluorescence of cells was analyzed on a Cytomics FC 500 MPL Flow Cytometer (Beckman Coulter, Brea, CA, USA). Cell cycle distributions were calculated using ModFit LT 2.0 software (Verity Software House, Topsham, ME, USA).

### Apoptosis assay

Cell preparation was performed as in the cell cycle experiments. An apoptosis assay was performed with the FITC Annexin V Apoptosis Detection Kit I (BD Biosciences, San Diego, CA, USA) according to manufacturer's protocol. Briefly, after treatments, cells were harvested, washed twice with cold PBS and resuspended in 100 μl of 1X binding buffer. Then, resuspended cells were incubated with 5 μl of FITC Annexin V and 5 μl of propidium iodide solution (50 μg/ml) for 15 min at room temperature in the dark. Finally, 400 μl of 1X binding buffer was added, and the cells were analyzed by flow cytometry within 1 h. The data represent both early (Annexin V-positive, PI negative) and late (Annexin V-positive, PI positive) apoptotic cells.

### Real time quantitative PCR

#### RNA isolation

Cells were plated in 10 cm dishes at a density of 5 × 10^5^ cells per dish and allowed to attach. After 48 h of treatment with EGFR inhibitors, cells were collected, and total RNA was extracted using an RNeasy Mini Kit according to the manufacturer's instructions (Qiagen, Crawley, UK). RNA was eluted in 40 μl of RNase-free water, and concentrations were determined using a NanoDrop ND-1000 spectrophotometer (NanoDrop Technologies Inc., Wilmington, DE, USA). The integrity of the RNA samples was assessed using an Agilent 2100 Bioanalyzer (Agilent Technologies, Foster City, CA, USA).

#### Reverse transcription

The cDNA was reverse transcribed from 1 μg total RNA in a 20 μl reaction volume using the High Capacity cDNA kit with RNAse inhibitor according to the manufacturer's instructions (Applied Biosystems, Foster City, CA, USA). Reaction conditions were 25°C for 10 min, 37°C for 120 min and 85°C for 5 min.

#### Taqman low density arrays (TLDA)

The expression of 43 genes involved in apoptosis, cell cycle control and coding for components of PI3K/AKT and RAS/MAPK pathway (Table 2) was quantified using custom-made TLDA, which are 384-well microfluidic cards preloaded with sets of primers and specific probes (Applied Biosystems, Foster City, CA, USA). Two genes were used as internal controls (GAPDH and 18S). cDNA samples were mixed with 2X Taqman Universal PCR Master Mix (Applied Biosystems), loaded onto the TLDA card and centrifuged twice for 1 min at 1200 rpm. Cards were sealed to prevent cross-contamination, and quantitative real time PCR amplification was performed using an ABI Prism 7900 HT Sequence Detection System according to the manufacturer's instructions (Applied Biosystems).

#### Relative quantification analysis

Threshold cycle (Ct) values were determined with RQ Manager 1.2 software (Applied Biosystems). We chose Ct > 35 as the cutoff for non-expressed genes. Each Ct value was normalized to the average Ct of two endogenous controls (GAPDH and 18S). The relative quantification (RQ) of gene expression was determined using the comparative ΔΔCt method based on the following equation: RQ = 2-ΔΔCt with ΔΔCt = ΔCt (treated cells) - ΔCt (untreated cells) and ΔCt = Ct (target gene) - Ct (endogenous gene) [78]. Unsupervised hierarchical clustering analysis based on ΔCt values was performed to identify differential gene expression profiles among the four cell lines. Gene expression profiles were clustered using Euclidean distance and Ward's method with SEM statistical software (Statistics Epidemiology Medicine) [79].

### Statistical analysis

All experiments were repeated at least 3 times, and the results are presented as the means ± SEM. Statistical significance between treated cells and untreated cells was evaluated using two-way analysis of variance (ANOVA) followed by an unpaired Student's t-test. A probability value p < 0.05 was considered significant.
